# Correspondence on “Needs Assessment for Updating Institute of Medicine Standards for Trustworthy Clinical Practice Guidelines”

**DOI:** 10.1002/gin2.70048

**Published:** 2025-09-20

**Authors:** Zijun Wang, Hui Liu, Yaolong Chen

**Affiliations:** ^1^ Research Unit of Evidence‐Based Evaluation and Guidelines, Chinese Academy of Medical Sciences (2021RU017), School of Basic‐Medical Sciences Lanzhou University Lanzhou Gansu China; ^2^ World Health Organization Collaboration Center for Guideline Implementation and Knowledge Translation Lanzhou Gansu China; ^3^ Institute of Health Data Science Lanzhou University Lanzhou Gansu China; ^4^ Key Laboratory of Evidence Based, Medicine of Gansu Province Lanzhou University Lanzhou Gansu China

Christopher Wolfkiel and colleagues have published a valuable needs assessment for updating the IOM standards [1]. This study is highly necessary and leading. However, we would like to offer several comments on the study's conclusions and provide suggestions regarding key areas for updating.

We fully agree with the necessity for updating the IOM standards and the identified key areas for revision in the article. Establishing an authoritative body to periodically assess and revise the standards is indeed a crucial first step. However, the article does not offer concrete strategies or implementation plans for the revision process. Given that the IOM standards are intended as overarching principles rather than a detailed handbook, the revision should aim to reflect the evolving healthcare environment and integrate contemporary theories and advances from related fields. In this context, we propose the following suggestions:

1. For conflict of interest (COI) management, Xun et al. [2] have developed a relatively comprehensive framework, which is included in the extended RIGHT (Reporting Items for practice Guidelines in HealThcare) checklist of COI&F.
2. To better incorporate and report stakeholder involvement in guideline development process, Xuan et al. [3] are currently developing the RIGHT‐MUSE checklist, an extension of RIGHT dedicated to stakeholder engagement.
3. On the topic of guideline certification, as raised in the article, Nan et al. [4] developed the STAR tool to evaluate the scientificity, transparency, and applicability of guidelines. Since 2023, the STAR Working Group has been conducting annual rankings of Chinese clinical guidelines, identifying trustworthy guidelines and supporting certification efforts.

Most of the above tools have attracted attention in the relevant fields and may serve as practical references for updating the IOM standards. Therefore, we strongly recommend the update of IOM standards should emphasize the integration and promotion of such mature, widely accepted tools.

Additionally, we think the urgency of the update may be overstated in the article's conclusions. While the general survey shows most respondents agree the standards “should be subject to review,” this indicates a need, but not necessarily urgency. Similarly, the domain‐ and item‐level analysis reveals that “Hold now” was the most selected option, suggesting that many elements are still viewed as currently appropriate. To enhance clarity and reduce potential reader confusion, we suggest defining a threshold for interpreting urgency. For example, if fewer than 80% of respondents select “Hold now,” an item could be considered for update. Such an approach would help align conclusions more closely with the presented data.

We hope these comments will be helpful in guiding future updates of the IOM standards.

## Conflicts of Interest

The authors declare no conflicts of interest.

## Data Availability

The authors have nothing to report.
